# Medical Cannabis Laws and Adolescent Alcohol Use Initiation

**DOI:** 10.26828/cannabis/2022.03.001

**Published:** 2021-11-21

**Authors:** Gregory A. Hard, Abenaa A. Jones, Abhery Das, Julie K. Johnson

**Affiliations:** 1 MGH Institute of Health Professions, 36 First Avenue, Boston MA 02129; 2 Division of Child and Adolescent Psychiatry, McLean Hospital, Belmont, MA 02478; 3 Department of Human Development and Family Studies, The Pennsylvania State University, 105 Health and Human Development Building, University Park, PA 16802, USA; 4 University of California, Irvine, 653 East Peltason Drive, Irvine, CA 92617; 5 Cannabis Policy Research Center of Excellence, Research Department, Cannabis Control Commission, Commonwealth of Massachusetts, 2 Washington Square, 2nd Floor, Worcester, MA 01604

**Keywords:** medical cannabis laws, alcohol initiation, adolescents, drug policy, cannabis

## Abstract

**Background.:**

The effects of medical cannabis laws (MCLs) on adolescent alcohol use remains unclear. Previous literature investigates alcohol consumption rather than alcohol initiation among adolescents, and does not examine the effect by sociodemographic characteristics and state-level dispensary status. We used population representative, state-level data to examine the relationship between MCLs and adolescent alcohol initiation.

**Methods.:**

Data for this study were derived from the Youth Risk Behavior Survey (YRBS), a nationally representative, cross-sectional school-based survey administered by the Centers for Disease Control (CDC) in odd-numbered years from 1991 to 2015. We used a difference-in-difference model to assess pre and post effects of state MCL enactment on adolescent alcohol use initiation. Logistic regression analyses assessed associations between MCLs and varying ages of initiation. We further stratified our results by race/ethnicity, gender, and dispensary status.

**Results.:**

Results from adjusted logistic regression models showed higher odds of initiating alcohol among adolescents in states without MCLs when compared to adolescents in states with MCLs (OR 1.37, [95% CI = 1.29, 1.44]). This effect was consistent across age, race/ethnicity, and gender groups. Reductions in self-report of alcohol initiation were also consistently found in multiple age strata (9–10, 11–12, and 13–14), though this finding did not reach conventional levels of statistical detection in all race/ethnicities.

**Conclusions.:**

Our findings support a substitutive effect, suggesting that adolescents in states with MCLs, as opposed to states without MCLs, may substitute cannabis for alcohol. Considering the evolving landscape of medical cannabis laws and the proliferation of state-level legalization laws, further research into the effects of such policies, such as adult-use cannabis laws, is warranted to further elucidate their effects on adolescent substance use.

As of August 2021, 36 states have approved medical cannabis laws (MCLs), permitting cannabis use with a medical evaluation for a wide range of clinical needs. The public perception of cannabis’ safety is changing, particularly among youth, likely due in part to changing cannabis laws across the United States (Hammond et al., 2020; Carliner et al., 2017). For instance, perceptions of the drug’s risks have been shown to be lower in MCL states than in non-MCL states (Keyes et al., 2016; Miech et al., 2016). The recent increase in state laws legalizing adult-use cannabis may further contribute to this trend (Cerdá et al., 2017). However, changing perceptions do not appear to translate into increased rates of cannabis use (Sarvet et al., 2018), with multiple analyses showing no relationship between the enactment of MCLs and increased rates of cannabis use (Johnson et al., 2017; Johnson et al., 2021; Melchior et al., 2019).

The effects of MCLs on the use of other substances by adolescents, including alcohol, remains unclear and a subject of debate. One analysis evaluated the effects of MCLs on the prevalence of past 30-day and 12-month alcohol use and report decreases in the rates of alcohol consumption among 8th graders after MCL enactment, with no effect noted in 10th or 12th graders (Cerdá et al., 2018). A study examining substance use behaviors in ten MCL states before and after implementation report no effect on alcohol consumption in 12–20 year olds (Wen et al., 2015). An analysis by Johnson and colleagues report modest reductions in the odds of past 30-day alcohol consumption and binge drinking behavior among adolescents in MCL states versus non-MCL states, including in states with less restrictive MCL provisions (Johnson, et al., 2017).

The data are mixed as to whether the relationship between alcohol and cannabis is complementary or substitutive, with some studies supporting substitution and others supporting complementarity. A literature review of 39 studies examining this relationship found that the liberalization of cannabis laws may decrease alcohol consumption, acting as a substitute, whereas environments with more restrictive alcohol policies may decrease cannabis use, thus acting as a complement (Subbaraman, 2016). An overlap in the usage patterns and trajectories between alcohol and cannabis has been noted in the literature (Nelson et al., 2015), with the use of both substances co-occurring. In one study of college students (O’Hara et al., 2016), authors report a direct relationship between alcohol consumption and the odds of cannabis use, suggesting complementarity. However, an inverse relationship was found in students who reported self-medicating with these substances, with reductions in alcohol consumption being associated with increases in cannabis use in this population, supporting a substitutive effect. A study in youth who were paid to abstain from cannabis for four weeks demonstrated an increase in the frequency of use and the amount of alcohol consumed during the abstinence period, further supporting a substitutive effect (Schuster et al., 2021).

Although the existing literature is illustrative, there are several limitations. First, much of the existing research examines alcohol consumption and alcohol use among adolescents, but does not evaluate the age of alcohol initiation. Earlier onset of alcohol initiation is considered to be a risk factor for the future development of alcohol and other substance use disorders (Bolland et al., 2013; Hawkins et al., 2015). Additionally, early initiation of alcohol is associated with adverse psychosocial outcomes, including an increased risk and earlier onset of major depression (Pedrelli et al., 2016; Rohde et al., 2001) and an increased risk of suicidal ideation and suicide attempts (Baiden et al., 2019; Bossarte & Swahn, 2011). Previous literature has not evaluated alcohol initiation across different races, ethnicities or genders. Research suggests that African American adolescents initiate alcohol later than white adolescents, and that female adolescents are more likely to continue using alcohol than their male counterparts (Malone et al., 2012). Investigating the association between MCLs and age of alcohol initiation allows for more a more effective deployment of substance use prevention resources. Further, the differential effects of alcohol initiation by age as well as race/ethnicity and gender may facilitate more targeted prevention strategies.

Given the possibility of a substitutive effect, we evaluated the relationship between MCLs and age of alcohol use initiation in adolescents. We examined 939,725 individuals in 46 states from 1991–2015, a period coinciding with the proliferation of MCLs. Given the spread of MCLs and the recent emergence of legalized adult-use cannabis in different states, it is essential to further elucidate the effects that such laws have on adolescent alcohol consumption and other substance use behaviors. Results from our study may hold particular relevance to understanding the ecology of MCLs as it relates to alcohol use initiation in adolescents.

## METHODS

### Population

Data for this study were derived from the state-level Youth Risk Behavior Survey (YRBS), which was established by the Centers for Disease Control and Prevention (CDC) to monitor the prevalence of health risk behaviors among adolescents (Brener et al., 2013). The YRBS is a representative, cross-sectional school-based survey administered to students in 9^th^ through 12^th^ grade in odd-numbered years from 1991 to 2015 across 46 states (N=939,725). Data from the remaining states (Oregon, Washington, Minnesota, and Hawaii) were unable to be obtained for varying reasons, including: application processes, lack of YRBS participation, or insufficient response rate.

The YRBS uses a two-stage cluster sampling design. Schools are randomly selected to participate, with the likelihood of selection being proportional to its enrollment. A random sample of classrooms are selected within each school, and all students within each class are asked to participate in the survey. Students complete the survey voluntarily during class, and parental permission is obtained in accordance with local school district policies. Data are weighted to adjust for nonresponse and the distribution of students by age, sex, and race/ethnicity in each school district. More information regarding the methodology of the YRBS is documented elsewhere (Brener et al., 2013).

### Measures

The primary exposure of interest in this analysis is the enactment of MCLs. Survey results in states with MCLs enacted at the time of data collection were compared to results from non-MCL states. The outcome of interest, the age of alcohol initiation as reported by survey respondents was assessed by the item: “How old were you when you had your first drink of alcohol other than a few sips?” The answer options included: “Never initiated Alcohol,” “8 years old or younger,” “9–10 years old,” “11–12 years old,” “13–14 years old,” “15–16 years old,” and “17 years old or older.” Adjustment variables included: state, year, and demographic variables (race/ethnicity, gender, and age). Control groups included all states without MCLs in any of the years of data collection and states without MCLs in any of the years of data collection combined with MCL states that did not enact MCLs by the year of data collection. Analyses additionally assessed whether states permit the operation of cannabis dispensaries in the enabling legislation and includes both active and inactive dispensary implementation (‘yes’, ‘no’), and whether states had dispensaries actively operating in the year of data collection (‘yes’, ‘no’).

### Data Analysis

Logistic regression models assessed associations between MCLs and varying ages of alcohol use initiation, race/ethnicity, and gender cohorts. Difference-in-difference (DiD) methodology assessed changes in alcohol use initiation among youth in states with and without medical cannabis laws pre-and-post MCL enactment. This econometric approach controls for external secular trends by using non-MCL state as a counterfactual. The DiD methodology is frequently used to assess the effects of cannabis legalization laws (Cerdá et al., 2017; Hasin et al., 2015) as well as in public health policy research more broadly (Dimick & Ryan, 2014). This approach assumes parallel trends in the outcome among the treatment and control groups prior to implementation of the policy. By visualizing outcomes over time, we confirmed that MCL and non-MCL states had parallel trends in the outcome prior to policy implementation. Stata 15 was used for all analyses. To account for the complex sampling design, Stata’s *svyset command* was used to assign YRBS design variables as provided by all state YRBS datasets (Brener et al., 2013). We used robust standard errors to adjust for heteroscedasticity in the residuals and any correlation of errors within the specified clusters (i.e., states). Johns Hopkins Bloomberg School of Public Health Institutional Review Board deemed this study exempt because it used publicly available, deidentified data.

## RESULTS

**Figure 1.**
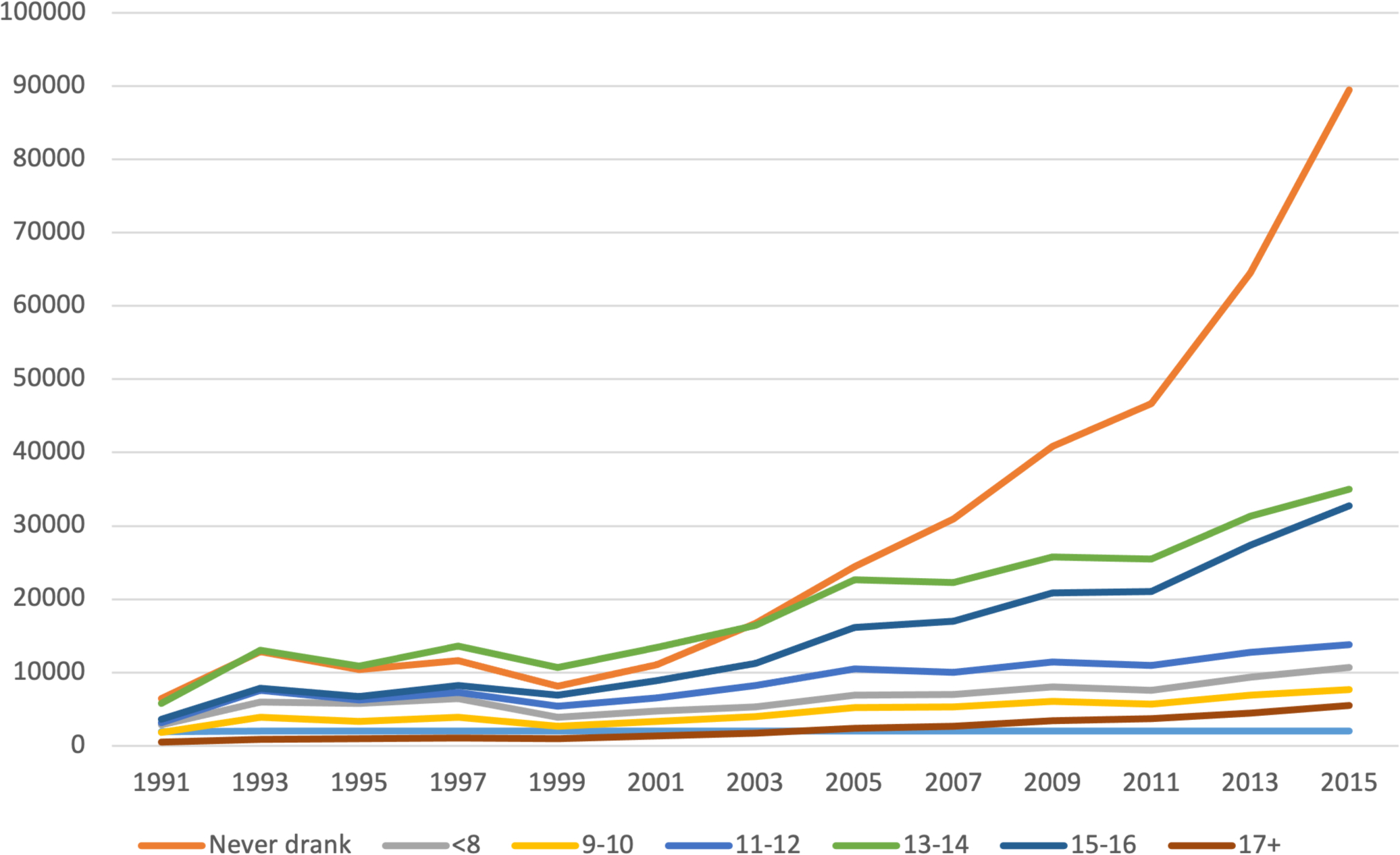
Alcohol Initiation by Age Group, 1991–2015

Table 1 contains the demographic composition of the study sample comprising White, Black, Hispanic, and other race/ethnicities, with the majority of adolescents ranging from 12–18 years old. Table 2 lists all the states included in our analysis, the year the MCL was enacted in that state and the number of years that were available for analysis both pre-enactment and post-enactment. Table 3 contains the number of participants in MCL versus non-MCL states from 2001–2015, biannually. Table 4 displays logistic regression results of adjusted odds ratios and their corresponding confidence intervals, stratified by gender, age, race/ethnicity, and state dispensary status. Figure 1 displayes the trends of alcohol initiation over time, stratified by age.

After adjusting for state effects, year effects, and demographic variables, there is a significant increase in the odds of initiating alcohol among adolescents in states without MCLs relative to states with MCLs (OR 1.37, [95% CI = 1.29, 1.44]). This general effect is consistent across all races and genders. Reductions in self-reported alcohol initiation are also consistently found in multiple age strata (9–10, 11–12, and 13–14), though this finding did not reach statistical significance in all race/ethnicities. Among specific age strata, the most significant reductions in self-reported age of alcohol initiation are noted among the 9–10 year old (OR 0.88, [95% CI = 0.83, 0.95]) and 11–12 year old cohorts (OR = 0.91, [95% CI = 0.86, 0.96]). A modest but statistically significant increase in alcohol initiation among the 15–16 year-old cohort was noted (OR = 1.06, [95% CI = 1.01, 1.12]). When stratified by race and gender, this effect only reaches significance for the 15–16 year-old female (OR = 1.12, [95% CI = 1.04 – 1.20]) and in Hispanic cohorts (OR = 1.13, [1.03 – 1.24]). This effect is not found among all males in the same age strata or among any of the other age or racial/ethnic strata. No differences were found in states with active dispensaries versus those without, across all strata.

## DISCUSSION

### Interpretation

This analysis, which used repeated cross-sectional data from 46 states in the Youth Risk Behavior Survey data, found a significant increase in the overall likelihood of alcohol initiation among adolescents residing in non-MCL states compared to those in MCL states. These results support a substitutive effect of alcohol initiation among youth in states that have enacted MCLs. This effect was fairly consistent across gender and race/ethnicity, though many age-stratified odds ratios did not reach statistical significance. There were no substantial differences noted among states with active dispensaries compared to those without. Further research is warranted to elucidate the relationship between cannabis access laws and the use of alcohol and other substances by youth. Continued monitoring is pertinent as cannabis laws continue to develop, evolve, and saturate across states.

### Implications and Future Directions

With the evolving nature of cannabis policy in the United States, ongoing research into the effects of such policies warrants further investigation regarding their effects on adolescent substance use and on public health more broadly. The continued Federal criminalization and classification of cannabis as a Schedule 1 drug under the Controlled Substances Act, combined with a patchwork of evolving state-level policies with varying restrictions and provisions, introduces a unique research challenges, leaving many gray areas for policy makers to navigate when drafting cannabis policies. These facts are also illustrative of the need for continuing, ongoing research that takes into account the dynamic and heterogeneous nature of these provisions. Future research efforts should examine the effects of adult-use cannabis laws, which could theoretically increase adolescent access to cannabis and subsequently affect other substance use behaviors, including alcohol. Given the relative novelty of adult-use cannabis laws and the evolving nature of these policies, regulations, and enforcement, and the overall changing political landscape, limited data exists on this topic and should be a target for future research.

### Limitations and Strengths

These results should be considered in the context of its limitations. We did not account for the enactment of additional policies that could also affect alcohol initiation, such as co- occurring state-level prevention efforts or the proliferation of adult-use cannabis laws across the U.S. starting in 2012, that this study was unable to obtain data for. We attempted to account for these differences in our analysis by using state and year fixed effects along with a difference-in-difference methodology to account for co-occurring trends across states and years (Angrist & Pischke, 2008). The YRBS data, which was the basis for this study, surveyed adolescents in public school settings. Although weighting was used to achieve samples that were representative of the state populations, these results may not be entirely generalizable to all adolescents, such as those who have dropped out, are home schooled, attend private or religious schools, or students who were placed in other alternative school settings. The YRBS relies on self-report, introducing the possibility of recall bias and social acceptance bias.

### Conclusion

The likelihood of alcohol initiation among adolescents is higher in states without MCLs compared to those with MCLs. These data support a substitutive effect, with adolescents potentially substituting alcohol for cannabis. With the proliferation of adult-use cannabis laws, future research should examine whether those laws are associated with decreases in alcohol initiation or increases in cannabis use among adolesceents. Due to the possibility of a substitutive effect, particularly in adolescents who self-medicate with cannabis, further research should examine the psychosocial and mental health impacts of increased cannabis consumption in this population. Further, analyses should adjust for cannabis policy heterogeneity, as differences in policy and regulation may have differential outcomes in youth cohorts.

## Figures and Tables

**Table 1.** Sample Characteristics (Race/Ethnicity, Gender, and Age) for Participants in the YRBS from 46 U.S. States, 1991–2015 (N = 939,725)

Demographics	N (%)
**Race**	
White^a^	574,454 (61.1%)
Black^a^	136,543 (14.5%)
Hispanic	132,783 (14.1%)
Other Race^a^	95,945 (10.2%)
**Age**	
12 years or younger	3,440 (0.37%)
13 years old	3,426 (0.36%)
14 years old	114,522 (12.19%)
15 years old	245,825 (26.16%)
16 years old	249,933 (26.60%)
17 years old	213,367 (22.71%)
18 years old or older	109,212 (11.62%)
**Gender**	
Male	461,593 (49.1%)
Female	478,132 (50.9%)

a Non-Hispanic

**Table 2.** States that Enacted MCLs Between 1991–2015 (Study Years)

State	Year MCL Enacted	Years of Data Pre-MCL Enactment	Years of Data Post-MCL Enactment
California (CA)	1996	0	1
Alaska (AK)	1998	1	5
Maine (ME)	1999	2	8
Colorado (CO)	2000	0	3
Nevada (NV)	2000	4	7
Montana (MT)	2004	6	6
Vermont (VT)	2004	0	3
Rhode Island (RI)	2006	4	5
New Mexico (NM)	2007	2	5
Michigan (MI)	2008	6	4
Arizona (AZ)	2010	4	3
New Jersey (NJ)	2010	3	2
Delaware (DE)	2011	5	2
Connecticut (CT)	2012	5	2
Massachusetts (MA)	2012	7	2
Illinois (IL)	2013	5	2
New Hampshire (NH)	2013	7	2
New York (NY)	2014	8	1
Maryland (MD)	2014	5	1

**Table 3.** Non-MCL and MCL State Samples, 2001–2015

Year	Non-MCL States N (%)	MCL States N (%)
2001	37,235 (93.0%)	2,815 (7.0%)
2003	62,414 (92.4%)	5,143 (7.6%)
2005	84,276 (88.9%)	10,502 (11.1%)
2007	81,124 (85.9%)	13,303 (14.1%)
2009	82,367 (71.8%)	32,324 (28.2%)
2011	83,267 (64.2%)	46,455 (35.8%)
2013	120,267 (72.5%)	45,557 (27.5%)
2015	48,357 (24.1%)	151,925 (75.9%)

**Table 4.** Adjusted Odds Ratios (ORs) for Adolescent Alcohol Initiation and State-MCL Enactment Status, 1991–2015 (N=939,725)

Age of Alcohol Initiation	Adjusted OR (95%CI)^a^
Never initiated Alcohol	1.37 (1.29– 1.44)***
8 years old or younger	1.00 (0.93– 1.07)
9–10 years old	0.88 (0.83– 0.95)***
11–12 years old	0.91 (0.86– 0.96)***
13–14 years old	0.94 (0.90–0.99)*
15–16 years old	1.06 (1.01– 1.12)*
17 years old or older	1.04 (0.95–1.14)
***Female***	
Never initiated Alcohol	1.36 (1.28–1.45)***
8 years old or younger	0.99 (0.91–1.08)
9–10 years old	0.85 (0.77–0.95)**
11–12 years old	0.89 (0.82– 0.97)**
13–14 years old	0.96 (0.92– 1.01)
15–16 years old	1.12 (1.04– 1.20)**
17 years old or older	1.00 (0.89–1.13)
***Male***	
Never initiated Alcohol	1.37 (1.29– 1.46)***
8 years old or younger	1.00 (0.92– 1.09)
9–10 years old	0.90 (0.83– 0.99)*
11–12 years old	0.92 (0.86–0.98)*
13–14 years old	0.93 (0.86–0.998)*
15–16 years old	1.00 (0.94– 1.07)
17 years old or older	1.08 (0.94– 1.24)
***White***	
Never initiated Alcohol	1.35 (1.26– 1.44)***
8 years old or younger	0.96 (0.87– 1.06)
9–10 years old	0.93 (0.84– 1.02)
11–12 years old	0.90 (0.84– 0.96)**
13–14 years old	0.96 (0.91– 1.02)
15–16 years old	1.04 (0.98– 1.12)
17 years old or older	1.09 (0.97– 1.22)
***Black***	
Never initiated Alcohol	1.37 (1.27– 1.48)***
8 years old or younger	0.98 (0.86–1.13)
9–10 years old	0.80 (0.69– 094)**
11–12 years old	0.84 (0.72– 0.98)
13–14 years old	0.89 (0.81– 0.99)*
15–16 years old	1.09 (0.95– 1.25)
17 years old or older	0.96 (0.74– 1.24)
***Hispanic***	
Never initiated Alcohol	1.34 (1.22– 1.46)***
8 years old or younger	1.08 (0.92– 1.27)
9–10 years old	0.89 (0.77– 1.02)
11–12 years old	1.06 (0.93– 1.20)
13–14 years old	0.96 (0.88–1.04)
15–16 years old	1.13 (1.03– 1.24)*
17 years old or older	0.99 (0.78– 1.25)
***Other Race***	
Never initiated Alcohol	1.46 (1.27– 1.68)***
8 years old or younger	1.07 (0.90– 1.28)
9–10 years old	0.88 (0.73– 1.06)
11–12 years old	0.93 (0.79– 1.10)
13–14 years old	0.88 (0.77–0.996)*
15–16 years old	1.06 (0.94– 1.20)
17 years old or older	1.03 (0.78– 1.37)
***Dispensaries Allowed***	
Never initiated Alcohol	1.27 (1.19– 1.35)***
8 years old or younger	1.08 (0.99– 1.18)
9–10 years old	0.88 (0.80– 0.95)**
11–12 years old	0.91 (0.85– 0.97)**
13–14 years old	0.95 (0.90– 0.997)*
15–16 years old	1.09 (1.02– 1.16)**
17 years old or older	1.05 (0.94– 1.17)
***Dispensaries Active 2015***	
Never initiated Alcohol	1.27 (1.20– 1.35)***
8 years old or younger	0.96 (0.89– 1.05)
9–10 years old	0.89 (0.82– 0.97)**
11–12 years old	0.90 (0.84– 0.97)**
13–14 years old	0.95 (0.91– 0.997)*
15–16 years old	1.07 (1.01– 1.13)*
17 years old or older	1.12 (1.00– 1.24)*

*Note*.

Estimates are weighted using YRBS weights [*p <.05, **p<.01, ***p<.001].

a Adjusted for: year, state, and individual demographics: age, gender, and race (White, Black, Hispanic, and Other).
